# Phenolic compounds isolated from fermented blueberry juice decrease hepatocellular glucose output and enhance muscle glucose uptake in cultured murine and human cells

**DOI:** 10.1186/s12906-017-1650-2

**Published:** 2017-03-04

**Authors:** Abir Nachar, Hoda M. Eid, Melinda Vinqvist-Tymchuk, Tri Vuong, Wilhelmina Kalt, Chantal Matar, Pierre S. Haddad

**Affiliations:** 1 Natural Health Products and Metabolic Diseases Laboratory, Department of Pharmacology and Physiology, Université de Montréal, Station Centre-Ville, P.O. Box 6128, Montréal, Québec H3C 3J7 Canada; 2 Canadian Institutes of Health Research Team in Aboriginal Antidiabetic Medicines and Montreal Diabetes Research Center, Montreal, Canada; 3 Department of Pharmacognosy, University of Beni-Suef, Beni-Suef, Egypt; 4 Food chemistry, Agriculture and Agri-Food Canada, Government of Canada, Kentville, Nova Scotia Canada; 5 Department of Nutritional Sciences, Faculty of Health Sciences, University of Ottawa, Ottawa, Canada

**Keywords:** Diabetes, Glucose homeostasis, Glucose-6-phosphatase, Glycogen Synthase, Glucose transport

## Abstract

**Background:**

We recently reported that blueberry juice fermented (FJ) with *Serratia vaccinii* bacterium has antidiabetic activities both in vivo and in vitro. The purpose of this project was to elucidate the effect of FJ on glucose homeostasis in liver and skeletal muscle cells and to identify active fractions/compounds responsible for this effect.

**Methods:**

FJ was fractionated using standard chromatography procedures. Hepatic (H4IIE, HepG2) and skeletal muscle cells (C2C12) were treated with maximum non-toxic concentrations of FJ, fractions and isolated compounds thereof. Glucose-6-phosphatase (G6Pase) activity was measured using glucose oxidase method. To measure glucose uptake and glycogen synthase (GS) activity, radioactive assays were used.

**Results:**

Fractionation of FJ yielded seven fractions. FJ and its phenolic fractions F2, F3-1 and F3-2 respectively inhibited G-6Pase by 31, 45, 51 and 26%; activated GS by 2.3-, 2.3-, 2.2- and 2-fold; and stimulated glucose uptake by 19, 25, 18 and 15%, as compared to DMSO vehicle control. Subfractionation of the active fractions yielded 4 compounds (catechol, chlorogenic, gallic and protocatechuic acid). Catechol, yielding the greatest bioactivity in G6Pase and glucose uptake assays, decreased G6Pase activity by 54%, increased GS by 2-fold and stimulated glucose uptake by 44% at 45.5 *μ*M.

**Conclusions:**

This study identifies novel potential antidiabetic compounds that can help standardize FJ.

**Electronic supplementary material:**

The online version of this article (doi:10.1186/s12906-017-1650-2) contains supplementary material, which is available to authorized users.

## Background

Type 2 diabetes (T2D) is a chronic metabolic disease that affects 382 million people worldwide and is associated with many complications, especially cardiovascular diseases [1]. Insulin resistance plays a major role in the physiopathology of type 2 diabetes. It is associated with an impaired insulin stimulation of glucose transport in muscle and fat as well as an impaired suppression of hepatic glucose production [2]. Nowadays, several people are using natural health products alone or in combination with their hypoglycemic drugs to manage their T2D. Currently, more than one third of Canadian diabetic patients are using alternative medicine [3, 4].

Members of the *Vaccinium* genus (family Ericaceae), notably *Vaccinium angustifolium* Ait (Canadian lowbush blueberry), are well known for their antidiabetic activities and have been used in the traditional medicine of many populations to treat T2D [5–8]. Previous studies have shown that different parts of the *V. angustifolium* plant reduces insulin resistance in obese rats [9] and possess insulin-like, glitazone-like and cytoprotective effects [10]. Blueberry fruits are rich in phenolic compounds with antidiabetic properties [8, 11]. Interestingly, the biotransformation of blueberry juice by a bacterium called *Serratia vaccinii* was found to greatly increase its content in phenolic compounds and its antioxidant activity [12].

This process also had an impact on the antidiabetic potential of blueberry juice. Indeed, fermented blueberry juice (FJ), in contrast to normal juice, stimulated glucose uptake in muscle cells and adipocytes using an insulin-independent pathway implicating the phosphorylation of AMP-activated protein kinase (AMPK) [13]. This antidiabetic effect was validated in an animal model using KK-Ay hyperphagic mice [14].

Glucose homeostasis results from equilibrium between the intestinal absorption of glucose, its production by the liver and its utilization by peripheral tissues such as muscle and fat [15]. Insulin regulates hepatic glucose production and storage. It inhibits some transcription factors like the forkhead family and the Peroxisome proliferator-activated receptor-gamma coactivator-1α (PGC-1α) leading to a decrease in the activity of Glucose-6-phosphatase (G6Pase), a key enzyme implicated in hepatic glucose production [16, 17]. On the other hand, insulin signaling phosphorylates glycogen synthase kinase-3 (GSK-3) leading to the activation of glycogen synthase (GS), a key enzyme implicated in glucose storage [18]. In addition, insulin regulates glucose uptake and utilization in muscle through the stimulation of glucose transporter 4 (GLUT4) translocation to the plasma membrane in order to mediate facilitative glucose diffusion [19].

In continuity with aforementioned studies on FJ in muscle cells and adipocytes, the aim of this project is to elucidate the antidiabetic action of FJ at the level of glucose homeostasis in cultured hepatocytes and muscle cells. Importantly, our purpose is to identify active fractions and compounds responsible of the antidiabetic activity of FJ.

## Methods

### Cell culture

All cell lines used – H4IIE (rat hepatoma), HepG2 (human hepatoma) and C2C12 (murine skeletal myoblasts) – were purchased from American Type Culture Collection (ATCC; Manassas, VA, USA). H4IIE cells were grown in a high glucose Dubelcco’s Modified Eagle Medium (DMEM) containing 10% Fetal Bovin Serum (FBS) and 0.5% antibiotics (PS: Penicillin 100 U/mL, Streptomycin 100 *μ*g/mL). HepG2 cells were grown in DMEM/F12 (50/50) medium, containing 10 FBS and 0.5% PS. On the other hand, C2C12 myocytes were cultured in DMEM medium containing 10 FBS, 10 HS and 0.5% PS then switched to DMEM medium containing 2% HS to initiate differentiation. Glucose uptake assay was performed on differentiated myotubes at the 7^th^ day of differentiation. All cells were cultured and incubated at 37 °C with 5% CO_2_ in 12-well plates for glucose uptake and G6Pase experiments and in 6-well plates for GS experiments. Overnight treatment (16–18 h) with the different samples was initiated prior to the determinations.

### Preparation of fermented blueberry juice

Mature lowbush blueberry fruits were purchased from Cherryfield Foods Inc. (Cherryfield, Maine, USA). The species was identified by the plant taxonomist Dr. Alain Cuerrier (Montreal Botanical Garden). The frozen fruit preparation represents a homogeneous mixture of several genotypes provided by many wild blueberry producers from Canada and Northeastern United States. We selected this starting material because it appropriately represents what is regularly used in the industry to prepare commercial wild blueberry juice. For the purposes of this study, blueberry fruit mixture (hereafter called juice) was prepared by blending the fruits (100 g) with an equivalent quantity (100 g) of Minimal Broth Davis without dextrose (MM) (Difco Laboratories, Detroit, MI, USA). The fruit mixture was then centrifuged to remove insoluble particles. The resulting juice was sterilized using 0.22 *μ*m Express Millipore filters (Millipore, Etobicoke, ON, Canada).

*Serratia vaccinii* were cultured as previously described [12]. The juice was inoculated with a saturated culture of *Serratia vaccinii* at (7.5 ± 0.3) log CFU ml − 1 corresponding to 2% of the total juice volume. A control flask was prepared under the same conditions but without inoculation. The blueberry preparations were incubated in a Lab-Line low-temperature benchtop incubated shaker (Lab-Line Instruments, Inc, Melrose Park, IL, USA) in 250-ml flasks at 22 °C, 120 rev min − 1, under aerobic conditions. After a 4 day fermentation period, the FJ was sterilized by 0.22 *μ*m filtration as detailed elsewhere [12].

In order to facilitate the handling and insure the stability of the blueberry preparations, they were freeze-dried to produce powdered material that was kept at −20 °C until use.

### Fractionation of FJ and identification of active compounds

**Fig. 1:**
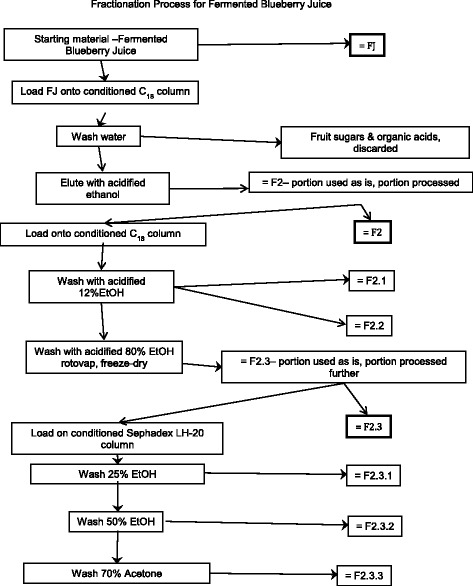
Fractionation Process for Fermented Blueberry Juice

Organic solvents were purchased from Fisher Scientific (Burlington, ON, Canada), unless otherwise mentioned. Normal blueberry juice has been partially characterized elsewhere [12, 13, 20, 21]. In the present studies, we carried out further phytochemical analysis using HPLC as described below. Figure 1 shows the fractionation scheme that was carried out to identify active compounds present in fermented blueberry juice (FJ). The starting material for the fractionation process was either normal blueberry juice prepared from wild blueberries (the control Juice, CJ), or the FJ in batches of approximately 500 ml. These were placed onto 29.5 cm × 5 cm chromatography columns (pre-conditioned with 1 column volume methanol then 2 column volumes water) containing Waters preparative C18 resin (125 Å, 55–105 *μ*) then washed with 2 column volumes of water, which was sufficient to remove sugars and organic acids (discarded). The phenolic compounds were eluted from the column using 1.2 column volumes of 13 mM trifluoroacetic acid (Sigma Aldrich, Oakville, ON) in ethanol. The ethanol eluent was dried using rotary evaporation and lyophilized to give the Phenolic fraction (F2). The CJ underwent no further fractionation after this initial step. On the other hand, a portion of F2 was dissolved in water and further fractionated on C18 column as outlined above. The first subfraction (F2.1) was eluted using 4 column volumes of 0.16 M HCl (Ricca Chemical Company, Texas, USA) and 2.06 M ethanol in water. This subfraction was composed mainly of gallic acid, protocatechuic acid, and catechol, as confirmed by HPLC (comparing retention times and UV-vis profiles of the peaks to standards). The next subfraction (F2.2) was eluted using an additional 2 column volumes of 0.16 M HCl and 2.06 M ethanol in water and was rich in chlorogenic acid (as confirmed by HPLC). The remaining bound materials, mainly flavonoids, were eluted using 0.16 M HCl and 13.7 M ethanol in water and were called the flavonoid fraction (F2.3). The three fractions were dried using rotary evaporation and lyophilisation. Next, a portion of the F2.3 fraction was dissolved in 4.28 M ethanol and applied to a 34.5 cm × 5 cm lipophilic Sephadex column (LH-20, 25–100 *μ*, Sigma Aldrich, Oakville, ON, Canada). HCl was neutralized and three fractions were collected. The first fraction enriched in anthocyanins (F2.3.1) was eluted using 7 column volumes of 4.28 M ethanol. The second fraction enriched in heteropolymers (F2.3.2) was then eluted using 3 column volumes of 8.56 M ethanol. Finally, a third fraction enriched in proanthocyanidins (F2.3.3) was eluted using 3 column volumes of 9.53 M acetone. All three fractions were dried using rotary evaporation and lyophilisation.

### HPLC analysis of normal and fermented blueberry juice

The HPLC system used was an Agilent 1100 binary pump system (Agilent Technologies, Mississauga, ON, Canada) with thermostatted column chamber, refrigerated autosampler, degasser, and DAD detection. Samples were separated on an Agilent Zorbax SB-C18 2.1×50mm 1.8 *μ* column held at 26 °C. Samples were held at 4 °C in the autosampler. Elution was done using a two solvent gradient (solvent A was 8 ml trifluoroacetic acid (TFA) per litre water (pH 1.35), solvent B was 6.8 ml TFA per liter acetonitrile) at a flow rate of 0.4 mL/min as follows: 5-10% B (0–12.5 min), 10–20% B (12.5–43.75 min), 20–100% B (43.75–45 min), hold 100% B 5 min, 100–5% B (50–51 min), re-equilibrating 9 min before the next injection. The following wavelengths were monitored: 280 nm (phenolics), 360 nm (flavonols), and 520 nm (anthocyanins), and as well full spectra were recorded from 190 to 600 nm. Designated pure compounds were quantified using authentic standards (Sigma Aldrich, Oakville, ON, Canada).

### Cytotoxicity assay (LDH)

The cytotoxicity assay was based on a lactate dehydrogenase (LDH) release kit (LDH Colorimetric Kit; Roche, Mannheim, Germany) and served to determine maximum non-toxic concentration of each sample in H4IIE, HepG2 and C2C12 cells. As was described previously [22], the cells were treated overnight (16–18 h) with CJ, FJ, fractions thereof, or pure compounds at different concentrations. The culture media were collected separately for each condition and kept on ice (representing released LDH from cells).

Then the cells were lysed by adding culture medium with 1% Triton, for 10 min at 37 °C, 5% CO_2_ (representing cellular LDH). All the samples were centrifuged at 250×*g*, 4 °C for 10 min and kept on ice in Eppendorf tubes. The ratio of released LDH to total LDH (total LDH = released LDH + cellular LDH) was calculated for each condition and results normalized to the values obtained from cells treated with the vehicle control (DMSO), always present at a final concentration of 0.1%. Maximum non-toxic concentrations for each sample were the highest ones that yielded LDH release comparable to that of DMSO controls.

### Glusose-6-phosphatase (G6Pase) activity

H4IIE cell line was used to measure the activity of G6Pase. Confluent cells were treated overnight (16–18 h) with DMSO 0.1% (vehicle control), insulin 100 nM (positive control), CJ, FJ, each of the seven fractions (at 5 *μ*g/mL) or each of the four pure compounds, all at their maximal non-toxic concentrations (Additional file 1: Table S1). After the treatment, cells were rinsed with PBS then lysed using a 15 mM phosphate buffer containing 0.05% Triton and 1.3 mM Phenol (pH = 6.5). A glucose-6-phosphate-containing buffer (200 mM) was then added to the cell lysates for 40 min at 37 °C; G-6-P contained in this buffer served as a substrate for endogenous G6Pase to yield glucose. A Wako AutoKit Glucose colorimetric assay (Wako Chemicals, Richmond, VA, USA) was used to determine the quantity of glucose generated in this reaction according to manufacturers’ recommendations. The BCA method was used to determine the protein content for each condition. Results were expressed relative to vehicle control (DMSO 0.1%).

### Glycogen Synthase (GS) activity

HepG2 cells were used to measure GS activity since this cell line exhibit a better expression of this enzyme compared to the H4IIE hepatocytes [23]. After achieving confluence in 6 well plates, the cells were treated overnight (16–18 h) with either vehicle control (DMSO 0.1%), the maximal non-toxic concentrations of CJ, FJ, each of the seven fractions (5 *μ*g/mL) or each of the four pure compounds (Additional file 1: Table S1). Treatment with insulin at 100 nM for 15 min was used as a positive control. After treatment, cells were rinsed with PBS then lysed in a buffer solution containing 50 mM glycylglycine, 100 mM sodium fluoride, 20 mM EDTA, 0.5% glycogen, pH 7.4 and a complete protease inhibitor cocktail added just before the assay. The lysates were centrifuged at 1000×*g* for 20 min at 4 °C. After centrifugation, 30 *μ*L of supernatant from each condition were added to 100 *μ*L of a specific buffer solution to measure active GS (25 mM glycylglycine, 0.275 mM UDP-glucose, 0.12 *μ*Ci/mL U-^14^C UDP-glucose, 1% glycogen, 1 mM EDTA, 10 mM sodium sulfate, pH 7.5) and another 30 *μ*L of supernatant were added to 100 *μ*L of a specific buffer solution to measure total GS (25 mM Tris, 5 mM UDP-glucose, 0.12 *μ*Ci/mL U-^14^C UDP-glucose, 1% glycogen, 3 mM EDTA, 5 mM glucose-6-phosphate, pH 7.9). The tubes were incubated in a water bath at 30 °C for 120 min. After incubation, 90 *μ*L of the mix for each condition was transferred on Whatman 31 ET chr 2 cm^2^ paper. The papers were rinsed with cold ethanol 66% (4 °C) for 30 min, then twice with ethanol 66% at room temperature for 30 min. After removing the ethanol, the papers were dipped in acetone for 2–3 min and dried. The papers were then transferred into scintillation vials. The radioactivity was measured using a liquid scintillation counter (LKB Wallac 1219; Perkin Elmer, Woodbridge, ON, Canada).

### Glucose uptake bioassay

C2C12 myocytes were grown in 12-well plates to 60% confluence then differentiated into myotubes over a 7-day period. On day 6 of differentiation, C2C12 cells were treated overnight (16–18 h) with either 0.1% DMSO (vehicle control), the maximal non-toxic concentrations of CJ, FJ, each of the seven fractions (12.5 *μ*g/mL) or each the four pure compounds (Additional file 1: Table S1). Metformin (400 *μ*M) was used as a positive control in similar conditions. After treatment, cells were rinsed twice with a warm Krebs phosphate buffer (KPB: 20 mM Hepes, 4.05 mM Na_2_HPO_4_, 0.95 mM NaH_2_PO_4_, 136 mM NaCl, 5 mM glucose, 4.7 mM KCl, 1 mM CaCl_2_, 1 mM MgSO_4_, pH 7.4) then incubated with KPB for 30 min at 37 °C. At this point, insulin (100 nM) was added to specific wells to act as another positive control (incubation in KPB buffer for 30 min). Cells were then rinsed twice with warm glucose-free KPB, then incubated in glucose-free KPB containing 0.5 *μ*Ci/mL 2-deoxy-D-^3^H-glucose (TRK-383, Amersham Biosciences, Baie d’Urfé, Canada) for 10 min at 37 °C. After incubation, cells were kept on ice and rinsed 3 times with ice-cold glucose-free KPB then lysed in 0.5 *μ*L of NaOH (0.1 M) for 30 min. The lysates were transferred with 1 mL of water to scintillation vials, then 4 mL of liquid scintillation cocktail (Beckman Coulter, Fullerton, USA) was added to each vial and incorporated radioactivity was measured using a liquid scintillation counter (LKB Wallac 1219; Perkin Elmer, Woodbridge, ON, Canada).

### Statistical analysis

All data were reported as the mean ± SEM of 3 different experiments with triplicates for each sample. Results were analyzed by one-way analysis of variance (ANOVA) (post-hoc pairwise comparisons were carried out with Bonferroni correction). In case the requirement of homogeneity of variances was not fulfilled, Games-Howell test for post-hoc was used. All statistical analyses were carried out using SPSS software, version 24 (IBM Corporation, NY, USA). A *p* value below 0.05 was considered statistically significant.

## Results

### Cytotoxicity assay (LDH)

After overnight treatment of H4IIE, HepG2 and differentiated C2C12 cells with CJ, FJ, each of the seven fractions or each of the four pure compounds at different concentrations, LDH released and total LDH were measured for each condition. Additional file 1: Table S1 shows the maximum non-toxic concentration determined for each sample and each cell line based on the results of LDH test.

### Fermented blueberry juice and its fractions decrease G6Pase activity and increase GS activation

**Fig. 2:**
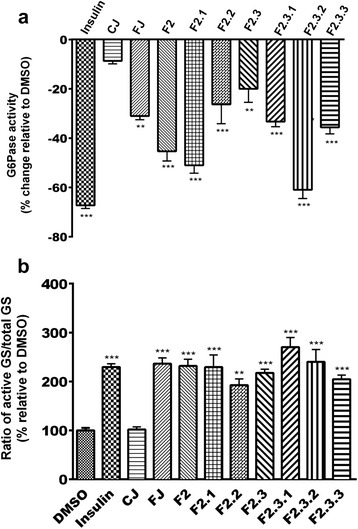
Effect of FJ and its seven fractions on G6Pase (**a**) and GS (**b**) activities. Results shown represent the change in G6Pase (**a**) and GS (**b**) activity observed after overnight treatment of H4IIE and HepG2 cells with maximum non-toxic concentration of control (CJ) and fermented (FJ) blueberry juice (both at 5 μg/mL) and the seven fractions yielded from FJ (at 5 μg/mL). Results are expressed relative to DMSO (0.1%) vehicle control (**a**: 0% inhibition; **b**: 100 % activity). Insulin (100 nM) was used as a positive control. **: *p*<0.01 and ***: *p*<0.001 significantly different from DMSO (n=3 in triplicates)

As illustrated in Fig. 2a, FJ significantly decreased G6Pase activity (31% reduction; *p* < 0.05) as compared to vehicle control (0.1% DMSO). This effect represented roughly half of the effect of the insulin positive control (67% decrease). In contrast, CJ did not induce a significant change in the activity of G6Pase. After fractionation of FJ, the seven fractions were tested in the G6Pase bioassay and all of them showed varied, yet statistically significant, inhibitory effect on the enzyme’s activity that ranged from 20 to 61% when compared to the DMSO (0% inhibition reference; Fig. 2a).

Figure 2b presents results concerning GS activity. FJ induced a significant increase in the enzyme’s activity (2.3 fold increase, *p* < 0.001) identical to that of the positive control, insulin (2.3 fold activation). As observed with G6Pase activity, CJ was without any significant effect. In addition, all seven fractions derived from FJ were able to significantly increase the activity of GS and ranged from 1.9- to 2.7-fold when compared to the DMSO vehicle control (100% activation; Fig. 2b).

### Phenolic fractions enhance glucose uptake in C2C12 muscle cells

**Fig. 3:**
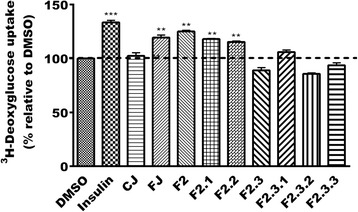
Effect of FJ and its seven fractions on 3H-deoxyglucose uptake. Results shown represent 3H-deoxyglucoseuptake by C2C12 cells after overnight treatment with maximum non-toxic concentration of control (CJ) and fermented (FJ) blueberry juice (both at 12.5 μg/mL) and the seven fractions yielded from FJ (at 12.5 μg/mL). Results are expressed as percentage relative to DMSO (0.1%) vehicle control. Insulin (100 nM) was used as a positive control. **: *p*<0.01 and ***: *p*<0.001 significantly different from DMSO (n=3 in triplicates)

When tested for their ability to enhance glucose uptake in C2C12 muscle cells, FJ showed a 20% stimulation of this uptake (*p* < 0.01) whereas CJ was without any effect (similar to vehicle control, DMSO 0.1%; Fig. 3). Insulin used as the positive control stimulated glucose transport into cells by 33% (*p* < 0.001). The phenolic fraction (F2) along with its subfractions F2.1 and F2.2, F2.3.2 were able to enhance glucose uptake into muscle cells by 25, 18 and 15% respectively (*p* < 0.01). Bonferroni post hoc test did not reveal significant differences between FJ, F2, F2.1, and F2.2. The other fractions were without effect when compared to vehicle control (100% activation; Fig. 3).

### Identification and isolation of phenolic compounds from the active fractions

**Fig. 4:**
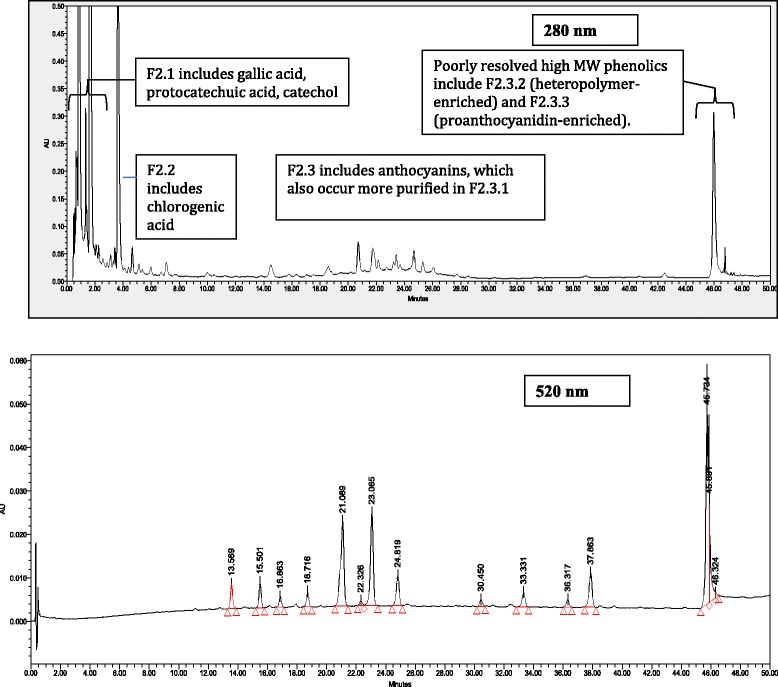
HPLC profile for the different fractions derived from Fermented Blueberry Juice (FJ)

Results of G6Pase and GS activity as well as glucose uptake bioassays were used to guide the identification of compounds found in the active fractions. HPLC analysis confirmed that the three fractions F2, F2.1 and F2.2 were composed mainly of four phenolic compounds: chlorogenic acid (CA), gallic acid (GA), protocatechuic acid (PA) and catechol (Cat). Compound identification was confirmed by comparing retention times and UV-vis profiles of each peak with respective standards (Fig. 4 and Additional file 1: Table S2). Calculation of phenolic compounds concentrations was based on the external standard method. The concentrations of individual phenolic compounds in FJ were as follows: CA (3.0 mg/g DW), GA (3.2 mg/g DW), PA (0.3 mg/g DW) and GA (3.2 mg/g DW).

### Identification of GA and Cat as the most active compounds acting on key enzymes implicated in hepatic glucose output

**Fig. 5:**
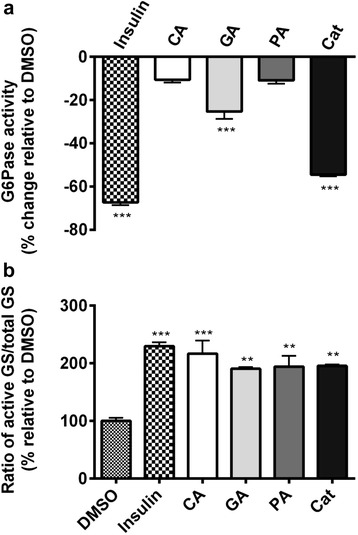
Effect of CA, GA, PA and Cat isolated from the active fractions of FJ on the activity of G6Pase (**a**) and GS (**b**). Results shown represent the change in G6Pase (**a**) and GS (**b**) activities observed after overnight treatment of H4IIE and HepG2 cells with maximum non-toxic concentration of isolated compounds: CA (70.5 μM), GA (147 μM), PA (162.2 μM) and Cat (45.5 μM). Results are expressed relative to DMSO (0.1%) vehicle control (**a**: 0% inhibition; **b**: 100 % activity). Insulin (100 nM) was used as a positive control. **: *p*<0.01 and ***: *p*<0.001 significantly different from DMSO (n=3 in triplicates)

As mentioned above, the most active fractions (F2, F2.1 and F2.2) were rich in phenolic compounds mainly CA, GA, PA and Cat. These compounds were tested at their maximal non-toxic concentration in the G6Pase and the GS assays. Two of the four compounds, namely CA and PA, did not significantly decrease G6Pase activity as compared to DMSO. In contrast, GA was able to reduce the enzyme activity by 25% (*p* < 0.05) whereas Cat yielded a 54% decrease in G6Pase activity (similar to the positive control, insulin) compared to the vehicle control DMSO 0.1% (*p* < 0.001, Fig. 5a). Games-Howell test showed that difference between Cat and GA was statistically significant (*p* < 0.05). In the GS assay, all of the four compounds were able to significantly increase the activity of the enzyme by about 2 fold (CA) or slightly less (GA, PA and Cat) when compared to DMSO 0.1% vehicle control (*p* < 0.05, Fig. 5b). These effects compared favorably with the effect of insulin (2.3 fold increase). No statistically significant differences were detected between the compounds.

### Enhancement of glucose uptake by CA, GA and Cat

**Fig. 6:**
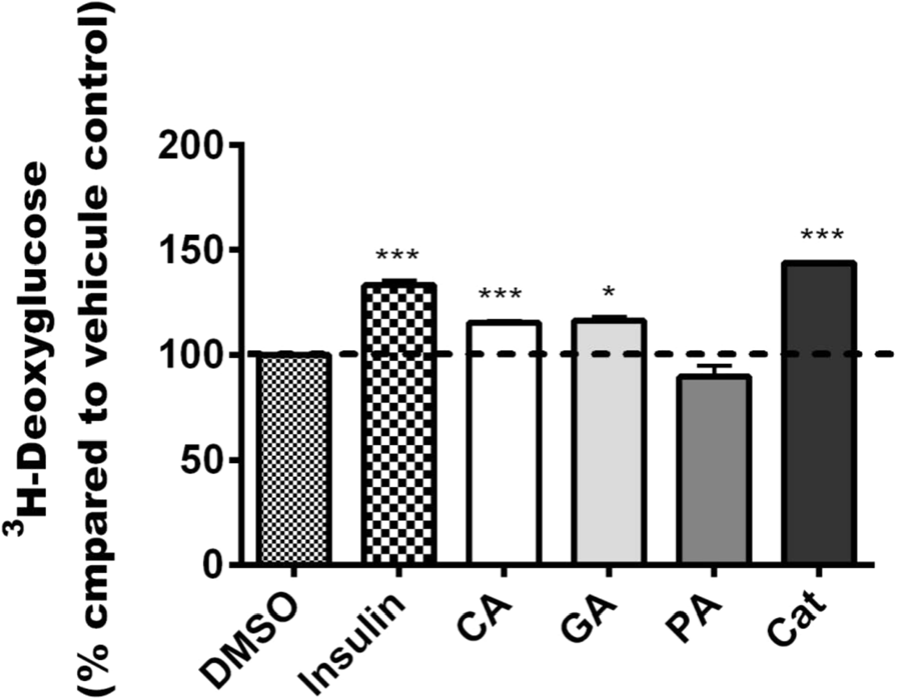
Effect of CA, GA, PA and Cat isolated from the active fractions of FJ on 3H-deoxyglucose uptake. Results shown represent 3H-deoxyglucose uptake by C2C12 cells after overnight treatment with maximum non-toxic concentration of isolated compounds: CA (70.5 μM), GA (147 μM), PA (162.2 μM) and Cat (45.5 μM). Results are expressed as percentage relative to DMSO (0.1%) vehicle control. Insulin (100 nM) was used as a positive control. *: *p*<0.05 and ***: *p*<0.001 significantly different from DMSO (n=3 in triplicates)

Along with their ability to regulate key enzymes implicated in hepatic glucose output, CA, GA and Cat significantly enhanced glucose uptake in C2C12 cells by 15% (*p* < 0,01), 16% (*p* < 0.05) and 43% (*p* < 0.001) respectively. In contrast, PA had no effect when compared to the vehicle control DMSO 0.1% (Fig. 6). Games-Howell test revealed that Cat yielded the greatest activity, it differed significantly from CA (*p* < 0.01) and GA (*p* < 0.05). CA and GA were not statistically different.

## Discussion

Insulin resistance is an important risk factor for the development of T2D. Along with the decrease in the phosphorylation of insulin receptor substrates (IRS-1 and IRS-2) and in PI3-K activity observed in insulin resistance, the translocation of glucose transporters and the activity of key enzymes implicated in glucose homeostasis are also reduced [24]. Despite the presence of many hypoglycemic drugs in the market, the control of glycaemia is sometimes hard to achieve. Moreover, populations in Canada and the USA use natural health products alone or in combination with their oral hypoglycemic in order to better manage diabetes [3, 4].

Several parts of the Canadian “lowbush blueberry” (*Vaccinium Angustifolium* Ait) plant were previously shown by our group to exert antidiabetic activities [10]. A recent study by Klimis-Zakas et al. has reported the ability of a wild blueberry-enriched diet to reduce metabolic syndrome risk factors such as chronic inflammation and endothelial dysfunction in obese Zucker rats, a rat model for metabolic dysfunction [25]. Meanwhile, Matar and colleagues demonstrated that biotransformation of blueberry juice by the *Serratia vaccinii* bacteria conferred a new phytochemical profile to the juice [12]. HPLC evaluation of the phenolic profiles of the normal and fermented juices detected differences in the contents of flavonoids and organic acids. Notably, gallic acid, which was not detected in normal juice, reached concentrations that varied from 26.7 ± 0.9 to 64.6 ± 0.5 mg/kg FW in FJ. On the other hand, chlorogenic acid was rather abundant in FJ, being present at a concentration of 852.7 ± 2.8 mg/kg FW. In general, total phenolic content nearly tripled after 3 days of fermentation (from 1251.3 ± 278.7 to 3640.3 ± 201.1 mg of gallic acid equivalent (GAE)/kg) and continued to increase modestly thereafter to reach 3926.3 ± 194.3 mg of GAE/kg after 7 days of fermentation [12]. Moreover, fermented blueberry juice (FJ) not only exhibited an increase in phenolic content but was also characterized by more pronounced antioxidant activities compared to the normal juice (CJ) [12, 13, 21]. Our laboratories therefore combined efforts to examine the impact of fermentation on the antidiabetic potential of wild blueberry preparations. We found that fermentation conferred to blueberry juice the capacity to enhance glucose uptake in muscle cells and adipocytes via phosphorylation of AMPK [13].

In the current studies, we first chose to further evaluate the antidiabetic potential of FJ by assessing rate-limiting enzymes of gluconeogenesis (G6Pase) and glycogen synthesis implicated in hepatic glucose output. Indeed, the regulation of hepatic glucose production, along with glucose utilization by peripheral tissues like muscle and fat, are key regulators of systemic glycaemia [17, 26]. We first compared effects of crude preparations of control and fermented blueberry juice. Consistent with our previous studies showing that fermentation conferred antidiabetic potential, we found that fermented blueberry juice significantly affected hepatocellular glucose homeostasis while control blueberry juice was without effect. Indeed, FJ significantly inhibited G6Pase (to a level roughly half as potent as insulin) and enhanced GS activity (to a level similar to insulin) in cultured murine and human hepatocytes, respectively. These results thus indicate that fermentation of blueberry juice also confers it the potential to control hepatic glucose output by reducing glucose production and increasing glucose storage. We also confirmed our previous finding in C2C12 cells [13] by demonstrating that our fermented blueberry preparation increased glucose transport whereas normal juice extract did not. This ascertained that the current lyophilized preparations behaved similarly to the actual juice that was used in these previous experiments.

Secondly and importantly, the current studies sought to isolate active fractions/compounds participating in the antidiabetic activity of the FJ in the muscle and liver cell bioassays of glucose homeostasis by using a phytochemical fractionation approach that we carried out previously to examine the protective effects of CJ on cardiomyocytes [27]. This approach yielded seven polyphenolic fractions of FJ, which were tested in the G6Pase, GS and glucose uptake bioassays. FJ fractions were able to significantly decrease G6Pase activity to levels ranging from 20 to 61%. The heteropolymer-enriched fraction, F2.3.2, gave the highest effect, which was very close to that of the positive control insulin. Unfortunately, the complexity of this fraction precluded further sub-fractionation. Next in apparent potency were the F2 and F2.1 fractions, the latter being enriched in GA, PA and Cat. In contrast, all FJ phenolic fractions increased the activity of GS within a relatively narrow range (1.9- to 2.7- fold increase), which was comparable to the action of the insulin positive control.

In terms of glucose uptake in skeletal muscle cells, on the other hand, significant glucose transport activity was associated only with three phenolic-enriched fractions, namely F2, F2.1 and F2.2. Since all fractions were active to varying degrees in both G6Pase and GS assays but only these three phenolic fractions showed a significant effect on glucose uptake, we examined in greater detail the constituents of the latter fractions. HPLC analysis revealed that the phenolic fractions contained mainly four compounds, namely chlorogenic acid (CA), gallic acid (GA), protochatecuic acid (PA) and catechol (Cat). These compounds are known for their antioxidant activity. Many studies also showed additional beneficial effects of CA, GA and PA. Indeed, CA has demonstrated anti-inflammatory, anti-diabetic, neuro-protective and cardio-protective properties [28–31]. GA exerts anti-diabetic activities [32], improves hyperglycaemia and glucose tolerance [33], while offering protection against diabetes complications, notably through cardio-protective properties [34, 35]. On the other hand, PA possesses anti-inflammatory [36, 37] and anti-apoptotic properties [38]. It also improves angiogenesis [39] and protects against hepatotoxicity and nephrotoxicity [40]. In contrast, and to our knowledge, no recent studies have addressed effects of catechol on glucose metabolism. Thus, our study is the first to reveal the effect of these phenolic compounds on hepatic glucose homeostasis and glucose transport in muscle.

The four compounds were able to increase GS activity to levels similar to those observed for FJ and its active fractions. GA and Cat showed stronger effects than the two others on the reduction of G6Pase activity. This effect was close to that observed for FJ and its active fractions. In terms of glucose transport, PA was inactive; GA and CA had similar effects, whereas Cat had the most pronounced activity. Interestingly, Cat stands out in this study as having the best activity profile in almost all bioassays, yielding equivalent if not stronger effects than FJ and its phenolic active fractions themselves.

## Conclusion

Altogether, the results of this study confirmed that fermentation of blueberry juice confers it antidiabetic potential in liver and skeletal muscle cells through the regulation of key hepatic enzymes implicated in gluconeogenesis and glycogen synthesis and the enhancement of skeletal muscle glucose uptake. Using a phytochemical fractionation approach, we now demonstrate that this activity resides principally in phenolic fractions and can be attributed, at least in part, to CA, GA, PA and Cat. This is congruent with studies showing antidiabetic properties for CA and GA, whereas it uncovers novel beneficial actions for PA. As mentioned, however, of all pure compounds in our study, Cat stood out as the most promising constituent, showing activity similar to or higher than the parent FJ (and even the insulin control) in all three bioassays. Future studies will be needed to further examine effects of flavonoids, anthocyanins, heteropolymers and proanthocyanins present in FJ.

Our study thus provides important insights into novel potential antidiabetic molecules that are produced when blueberry juice is fermented with *Serratia vaccinii*. Importantly, the identified compounds represent quality control tools that can be used to ensure the efficacy of FJ and hence standardize FJ preparations.

## Additional file

Additional file 1: Table S1. A. Optimum non-toxic concentrations of CJ, FJ and corresponding fractions used for bioassays in H4IIE, HepG2 and C2C12 cells. B. Maximum non-toxic concentrations of pure compounds used for bioassays in H4IIE, HepG2 and C2C12 cells. **Table S2.** Fractionation of fermented blueberry extract, indicating the major component(s) in each fraction (F). (DOCX 19 kb)

## Abbreviations

AMPK: AMP- activated protein kinase
Antho: Anthocyanins fraction
CA: Chlorogenic acid
Cat: Catechol
CJ: Control blueberry juice
FJ: Fermented blueberry juice
Flv: Flavonoids fraction
G6Pase: Glucose-6-Phosphatase
GA: Gallic acid
GLUT4: Glucose transporter 4
GS: Glycogen Synthase
GSK-3: Glycogen Synthase kinase-3
Hetero: Heteropolymers fraction
IRS: Insulin Receptor Substrate
PA: Protocatechuic acid
PAC: Proanthocyanidins fraction
PGC-1α: Peroxisome proliferator-activated receptor gamma coactivator 1-alpha
Phe: Phenolic fraction
