# Supplementary material for: Phenolic compounds isolated from fermented blueberry juice decrease hepatocellular glucose output and enhance muscle glucose uptake in cultured murine and human cells
Source: BMC Complement Altern Med. 2017 Mar 4;17:138. doi: 10.1186/s12906-017-1650-2 (PMC5336672; doi:10.1186/s12906-017-1650-2)
Supplement: Additional file 1: Table S1. — A. Optimum non-toxic concentrations of CJ, FJ and corresponding fractions used for bioassays in H4IIE, HepG2 and C2C12 cells. B. Maximum non-toxic concentrations of pure compounds used for bioassays in H4IIE, HepG2 and C2C12 cells. Table S2. Fractionation of fermented blueberry extract, indicating the major component(s) in each fraction (F). (DOCX 19 kb) [file 12906_2017_1650_MOESM1_ESM.docx]

**Table S1. A. Optimum non-toxic concentrations of CJ, FJ and corresponding fractions used for bioassays in H4IIE, HepG2 and C2C12 cells. B. Maximum non-toxic concentrations of pure compounds used for bioassays in H4IIE, HepG2 and C2C12 cells.**

| **A**  **Sample** | **H4IIE** | **HepG2** | **C2C12** |
| --- | --- | --- | --- |
| **Control Juice (CJ)** | 5 *μ*g/mL | 5 *μ*g/mL | 12.5 *μ*g/mL |
| **Fermented Juice (FJ)** | 5 *μ*g/mL | 5 *μ*g/mL | 12.5 *μ*g/mL |
| **Phe fraction (F2)** | 5 *μ*g/mL | 5 *μ*g/mL | 12.5 *μ*g/mL |
| **Early #1 fraction (F2.1)** | 5 *μ*g/mL | 5 *μ*g/mL | 12.5 *μ*g/mL |
| **Early #2 fraction (F2.2)** | 5 *μ*g/mL | 5 *μ*g/mL | 12.5 *μ*g/mL |
| **Flv fraction (F2.3)** | 5 *μ*g/mL | 5 *μ*g/mL | 12.5 *μ*g/mL |
| **Antho fraction (F2.3.1)** | 5 *μ*g/mL | 5 *μ*g/mL | 12.5 *μ*g/mL |
| **Hetero fraction (F2.3.2)** | 5 *μ*g/mL | 5 *μ*g/mL | 12.5 *μ*g/mL |
| **Proantho fraction (F2.3.3)** | 5 *μ*g/mL | 5 *μ*g/mL | 12.5 *μ*g/mL |
| **B**  **Sample** | **H4IIE** | **HepG2** | **C2C12** |
| **Chlorogenic acid (CA)** | 70.5 *μ*M | 70.5 *μ*M | 70.5 *μ*M |
| **Gallic acid (GA)** | 147 *μ*M | 147 *μ*M | 147 *μ*M |
| **Protocatechuic acid (PA)** | 162.2 *μ*M | 162.2 *μ*M | 162.2 *μ*M |
| **Catechol (Cat)** | 45.5 *μ*M | 45.5 *μ*M | 45.5 *μ*M |

**Table S2. Fractionation of fermented blueberry extract, indicating the major component(s) in each fraction (F).**

| Fraction | Major Component(s) | Starting Material | Column Resin | Eluant |
| --- | --- | --- | --- | --- |
| FJ | Sugars, organic acids, growth media, phenolics |  | C_18_ | Water |
| F2 | Phenolics | FJ | C_18_ | EtOH |
| F2.1 | Gallic acid, catechol, protocatechuic acid | F2 | C_18_ | 12% EtOH |
| F2.2 | Chlorogenic acid | F2 | C_18_ | 12% EtOH |
| F2.3 | Flavonoids | F2 | C_18_ | 80% EtOH |
| F2.3.1 | Anthocyanins | F3-3 | LH20 | 25% EtOH |
| F2.3.2 | Heteropolymers | F3-3 | LH20 | 50% EtOH |
| F2.3.3 | Proanthocyanidins | F3-3 | LH20 | 70% Acetone |
